# Negative regulation of fibroblast growth factor 10 (FGF-10) by polyoma enhancer activator 3 (PEA3)

**DOI:** 10.1016/j.ejcb.2009.01.004

**Published:** 2009-07

**Authors:** Athina-Myrto Chioni, Richard Grose

**Affiliations:** Centre for Tumour Biology, Institute of Cancer, Barts & The London School of Medicine & Dentistry, London EC1M 6BQ, UK

**Keywords:** FGF-10, PEA3, Breast cancer, Cell migration

## Abstract

FGF-10 plays an important role in development and disease, acting as the key ligand for FGFR2B to regulate cell proliferation, migration and differentiation. Aberrant FGF signalling is implicated in tumourigenesis, with several cancer studies reporting FGF-10 or FGFR2B upregulation or identifying activating mutations in *Fgfr2*. We used 5’ RACE to identify a novel transcription start site for murine *Fgf-10*. Conventional in silico analysis predicted multiple binding sites for the transcription factor PEA3 upstream of this site. Binding was confirmed by chromatin immunopreciptation, and functional significance was studied by both RNAi knockdown and transient over-expression of PEA3. Knockdown of PEA3 message led to increased *Fgf-10* expression, whereas overexpression of PEA3 resulted in decreased *Fgf-10* expression. Thus, we have identified PEA3 as a negative regulator of *Fgf-10* expression in a murine cell line and confirmed that activity also is seen in human breast cancer cell lines (MCF-7 and MDA-MB-231). Furthermore, over-expression of PEA3 in these cells resulted in impaired cell migration, which was rescued by treatment with FGF-10. Thus, PEA3 can regulate the transcription of *Fgf-10* and such modulation can control breast cancer cell behaviour.

## Introduction

Fibroblast growth factors (FGFs) comprise a family of 22 members that play important roles during embryogenesis and adulthood, regulating a wide range of cellular behaviours including proliferation, migration, survival and differentiation (Ornitz and Itoh, 2001). Genetic studies have shown that FGF-10, a secreted glycoprotein that acts as a paracrine signalling molecule in many tissues (Beer et al., 1997), is crucial for epithelial-mesenchymal interactions during development (Min et al., 1998; Sekine et al., 1999). FGF-10 signals through two transmembrane tyrosine kinase receptors – FGFR1B and FGFR2B, to which it binds with low and high affinities, respectively (Zhang et al., 2006). Genetic studies in mice have shown FGFR2B to be the key receptor for transducing FGF-10 signalling (Celli et al., 1998; De Moerlooze et al., 2000; Min et al., 1998; Sekine et al., 1999).

Aberrant FGF signalling has been linked not only with developmental abnormalities but also with cancer. Hypomorphic *Fgf-10* mutations have been shown to cause lacrimo-auriculo-dento-digital (LADD) syndrome-like defects in both mice and humans (Rohmann et al., 2006; Shams et al., 2007), and FGF-10 has been implicated in the development of craniosynostosis (Ibrahimi et al., 2004; Wilkie et al., 2002). Increased expression of FGF-10 has been described in several tumours, including those of the colorectum, prostate and breast (Matsuike et al., 2001; Memarzadeh et al., 2007; Nomura et al., 2008; Theodorou et al., 2004). Likewise, FGFR2B has been implicated in cancer susceptibility and progression in a variety of ways (Grose and Dickson, 2005; Katoh, 2008). Elevated expression of FGFR2B has been described in breast, colorectal, cervical, pancreatic and prostate cancers (Kurban et al., 2004; Matsuike et al., 2001; Memarzadeh et al., 2007; Meyer et al., 2008; Nomura et al., 2008). Activating mutations or amplifications of *Fgfr2* also have been identified in breast, lung, stomach and endometrial cancers (Adnane et al., 1991; Davies et al., 2005; Jang et al., 2001; Pollock et al., 2007). Furthermore, genome-wide single-nucleotide polymorphism (SNP) analyses have identified SNPs in FGFR2 that result in increased susceptibility to breast cancer by elevating FGFR2 expression (Easton et al., 2007; Hunter et al., 2007; Meyer et al., 2008). However, although FGFR2 signalling clearly plays an oncogenic role in some cancers, in several tissues, including bladder, skin and prostate, it also can act as a tumour suppressor (Feng et al., 1997; Grose et al., 2007; Ricol et al., 1999).

Polyoma enhancer activator 3 (PEA3), a member of the PEA3 family of ETS-family transcription factors (Sharrocks, 2001), also has been reported to play both oncogenic and tumour-suppressive roles in cancer. PEA3 expression has been shown to exert anti-proliferative effects on breast and ovarian cancer cells, and also to improve survival in mouse models of cancer (Xing et al., 2000; Yu et al., 2006). However, there are also many studies implicating PEA3 as a driving factor in several neoplasms, including breast, colorectal and lung and ovarian cancer (Benz et al., 1997; Davidson et al., 2003; Hiroumi et al., 2001; Liu et al., 2004).

PEA3 family members are expressed at many sites of epithelial-mesenchymal interaction during development (Chotteau-Lelievre et al., 1997). *Pea3* has been identified as a target of FGF-10/FGFR2B signalling in the developing lung, where its expression is induced in distal lung bud epithelial cells in response to a mesenchymally-derived FGF-10 signal (Liu et al., 2003), and also in the pancreas (Kobberup et al., 2007). Thus, although FGF-10 is known to regulate *Pea3* expression, our study is the first to describe the converse interaction; that is that PEA3 can regulate the expression of *Fgf-10*.

Despite a previous in silico study having predicted the transcription start site of murine *Fgf-10* (Katoh and Katoh, 2005), no experimental evidence exists to support this location. Since FGF-10 signalling provides a powerful regulatory signal, both in development and cancer, the aim of our present study was to identify a definitive transcription start site for *Fgf-10* in order to investigate possible regulatory mechanisms that may control its expression.

## Materials and methods

### Cell culture

MDA-MB-231 and MCF-7 cells were grown in Dulbecco's modified Eagle's medium (DMEM) without phenol red (Sigma, Poole, UK) supplemented with 10% foetal bovine serum (FBS; Biosera, Ringmer, UK) and 4 mM L-glutamine (CR-UK LRI Cell Services, Potters Bar, UK). Immortalised mouse endothelial cells, generated from an H-2KbtsA58 transgenic mouse line (Jat et al., 1991), were a kind gift from Dr. Gabi D’Amico Lago and Dr. Kairbaan Hodivala-Dilke. Endothelial cells were seeded into T75 Nunc tissue culture flasks (VWR, Lutterworth, UK) pre-coated for 1 h at 37 °C with 0.1% gelatine (Sigma), 10 μg/ml fibronectin (Sigma) and 30 μg/ml collagen (type I; PureCol^®^) (Inamed Biomaterials, Nutacon, The Netherlands). Cells were grown in low-glucose DMEM:Hams F-12 (1:1; CR-UK Cell Services), supplemented with 10% FBS, 4 mM L-glutamine, 20 U/ml IFN-γ (Peprotech, London, UK), 0.05 mg/ml endothelial mitogen (Biogenesis, Poole, UK) and 0.01 mg/ml heparin (Sigma). All cells were incubated at 37 °C, 8% CO_2_ and 100% relative humidity.

### RNA ligase-mediated rapid amplification of cDNA ends (RLM-RACE)

RLM-RACE was performed for the identification of the murine *Fgf-10* transcription start site. A FirstChoice^®^ RACE-ready cDNA kit was used according to the manufacturer's instructions (Ambion, Warrington, UK). Briefly, total RNA was isolated from E18.5 mouse lung using TriReagent (Sigma) and 10 μg was used for 5’ RLM-RACE, to facilitate the cloning of the full-length *Fgf-10* 5’ cDNA sequence. 5’ RLM-RACE adapter sequence is given in Table 1. Taq-amplified 5’ RLM-RACE PCR products were cloned into pBluescript SK^−^ vector (Stratagene, Cheshire, UK), and four clones were sequenced fully. The putative promoter region of murine *Fgf-10* (the 500 bp directly upstream of the newly identified transription start site) was analysed in silico using the ‘Transcription Element Search System’ (TESS) (Schug, 2003).

### Nested PCR for 5’ RLM-RACE

Nested PCR was performed according to the manufacturer's instructions (FirstChoice^®^ RACE-ready cDNA kit, Ambion). Briefly, 1 μl of the reverse transcriptase reaction was used for the outer 5’ RLM-RACE PCR, and 1 μl from the first PCR was used for the subsequent inner 5’ RLM-RACE PCR. Primer sequences are given in Table 1. Outer and inner PCRs were performed as follows: 3 min at 94 °C; 35 cycles (94 °C for 30 s, 60 °C for 30 s, 72 °C for 30 s); 72 °C for 7 min. Results were analysed by electrophoresis on a 2% agarose/TBE gel.

### Chromatin immunoprecipitation (ChIP) analysis

ChIP was performed, according to the manufacturer's instructions (ChIP kit; Upstate Biotechnology, Dundee, UK), to identify a possible interaction between the transcription factor PEA3 and the *Fgf-10* promoter. Briefly, immortalised mouse endothelial cells were used as starting material, with chromatin cross-linked by fixation in 1% formaldehyde for 10 min at 37 °C prior to lysing the cells. Approximately 1×10^6^ cells were lysed in 200 μl SDS lysis buffer (Upstate Biotechnology), and the was DNA sheared by sonication (4×10 s sonication; 6 μm amplitude with a 30-s break between each round; Soniprep 150 MSE, Sanyo, Japan) to an average length of approximately 500 bp. The range of DNA fragment size was confirmed by reversing the crosslinks with 8 μl 5 M NaCl in 200 μl cell lysate at 65 °C for 4 h, recovering the DNA by phenol/chlorophorm extraction and electrophoresing the sample on a 1% agarose/TBE gel). The sonicated cell lysate was diluted 10-fold in ChIP dilution buffer (Upstate), and 1% of the diluted DNA was kept as the input control for subsequent PCR analysis. Histones were pre-cleaned with salmon sperm DNA/protein A agarose 50% slurry for 1 h at 4 °C with agitation. Pre-cleaned chromatin was immunoprecipitated with 2 μg anti-PEA3 antibody (Santa Cruz, Heidelberg, Germany), anti-RNA polymerase II antibody (positive control; Upstate Biotechnology) or IgG1 control (BD Pharmingen, Oxford, UK) per sample overnight at 4 °C with constant rotation. The antibody/histone complex was collected by adding salmon sperm DNA/protein A agarose slurry (30% of the total sample volume) for 1 h at 4 °C with constant rotation. The samples were washed and eluted according to the manufacturer's instructions. Both the immunoprecipitated and the control input samples (each in 500 μl volume) were then subjected to cross-link reversal. To each sample was added; 10 μl 0.5 M EDTA, 20 μl 1 M Tris-HCl, pH 6.5, and 2 μl of 10 mg/ml proteinase K, and samples were incubated for 1 h at 45 °C. DNA was recovered by phenol/chloroform extraction and ethanol precipitation. PCR was performed with *Fgf-10* and *GAPDH* (positive control) primers (as used above).

### RNA interference

Cells (40–50% confluent in 6-well plates) were transfected for 4 h with 100 nM siRNA (Genome smart pool for mouse *Pea3* ETV4, NM_008815; D-001206-13-05; Dharmacon, Cramlington, UK) using Oligofectamine (Invitrogen, Paisley, UK). mRNA, protein level and functional activity were assayed 24–72 h post-transfection, and compared with mock- and/or control siRNA-treated cells (SiControl non-targeting siRNA pool; D-001210-02-20, Dharmacon). Transfection efficiency was assessed independently using a positive control siRNA lamin A/C (D-001050-01-05, Dharmacon) which was compared with the SiControl non-targeting siRNA.

### Cloning PEA3-encoding cDNA and transient transfection

Forward and reverse PCR primers containing engineered BamHI and XbaI recognition sites, respectively (bold in primer sequences given in Table 1), were used to amplify the full-length coding sequence of PEA3 from total RNA isolated from immortalised human keratinocytes (HaCaT cell line; kind gift of Prof Petra Boukamp (Boukamp et al., 1988)). The primers were used at an annealing temperature of 60 °C. The hPEA3 PCR product was cloned into the BamHI and XbaI sites of the pcDNA4/TO mammalian expression vector (Invitrogen).

MDA-MB-231 and MCF-7 cells were transfected with 1 μg DNA using 3 μl Lipofectamine 2000 (Invitrogen) for 4 h. Transfection efficiency of both cell lines was >85%, as determined by immunofluorescence imaging following transfection with a control pcDNA4/TO-GFP vector expressing green fluorescent protein (data not shown).

### RT-PCR and real-time RT-PCR

Extraction of total RNA was performed using an RNeasy kit (Qiagen, Crawley, UK). All samples were treated with DNase prior to synthesis of cDNA using Superscript II^®^ Reverse Transcriptase (Invitrogen). RT-PCR was performed using Megamix Blue^®^ PCR mastermix (Cambio, Cambridge, UK). Real-time RT-PCR was performed using a QuantiTech SYBR Green RT-PCR kit (Qiagen) with hypoxanthine-guanine phosphoribosyltransferase (*HPRT*) and glyceraldehyde 3-phosphate dehydrogenase (*GAPDH*) as housekeeping genes for normalisation. For real-time RT-PCR the threshold amplification cycles were determined using a 7900HT Fast Real-Time PCR machine (Applied Biosystems, Warrington, UK).

The primer pair sequences are given in Table 1. All primers were used at an annealing temperature of 60 °C.

### Western blotting

Cell lysates were prepared using RIPA buffer (Upstate Biotechnology) containing 1 mM NaF, 1 μg/ml aprotinin, 1 μg/ml leupeptin, 1 μg/ml pepstatin, 1 mM phenylmethylsulphonyl fluoride (PMSF) (all Sigma) and the protein yield was determined using a Bradford dye binding assay (Bio-Rad, Hercules, USA). Equivalent amounts of protein from different lysate samples (60 μg/well) were resolved by pre-cast 4–12% NuPAGE^®^ Novex^®^ Bis-Tris mini Gels (Invitrogen) gel electrophoresis. Four primary antibodies were used: anti-alpha-tubulin antibody (1:2000; Sigma; reactivity: mouse and human); anti-FGF-10 antibody (1:1000; Abcam, Cambridge, UK; reactivity: mouse and human); anti-PEA3 (1:500; Santa Cruz Biotechnology; reactivity: mouse, human, rat); anti-lamin A/C antibody (0.5 μg/ml; Santa Cruz Biotechnology; reactivity: mouse and human). Blots were developed with ECL (GE Healthcare, Amersham, UK). Densitometric analysis was performed using Image-Pro Plus software (Media Cybernetics, Bethesda, USA). Signal density was normalised to anti-alpha-tubulin antibody as a loading control/reference, for at least three separate treatments.

### Migration assay

Cells (1×10^5^) were plated onto 8 μm-pore Transwell^®^ migration filters in 24-well plates (Corning, New York, USA). PEA3- and/or empty vector-transfected cells were used 36 h post-transfection. Cells were incubated either in DMEM supplemented with 4 mM L-glutamine and 1–10% FBS gradient or in DMEM supplemented with 0.1% BSA (Sigma) and with 4 mM L-glutamine without FBS overnight (12 h). Where mentioned, recombinant FGF-10 (100 ng/ml; Peprotech, London, UK) together with heparin (300 ng/ml; Sigma) was added to the serum-free medium. When an FBS gradient was used, the bottom of the Transwell^®^ membrane was pre-coated with 0.01% poly- L-lysine solution (Sigma) for 1 h at room temperature prior to plating the cells. In the absence of FBS, the bottom of the Transwell^®^ was coated with 10 μg/ml fibronectin (Sigma) for 1 h at 37 °C prior to plating the cells. Cells from the upper chamber, which had not migrated, were removed with a cotton bud. Cells that had migrated to the lower surface were trypsinised and the cell number was determined using an automatic CASY cell counter (Scharfe system, Leipzig, Germany).

### Proliferation assay

PEA3 and/or empty vector-transfected cells (5×10^4^) were plated in a 24-well plate in DMEM supplemented with 4 mM L-glutamine and 10% FBS. Cells were trypsinised and counted after 12 h using an automatic CASY cell counter.

### Data analysis

All quantitative data are presented as means±standard errors, unless stated otherwise. Statistical significance was determined with Student's *t*-test or Mann-Whitney Rank Sum test as appropriate. When multiple conditions were compared, one way analysis of variance (ANOVA) followed by Newman-Keuls test was performed. Real-time PCR data were analysed using the 2^−ΔΔCt^ method (Livak and Schmittgen, 2001). Results were considered significant at P<0.05 (*). Unless otherwise stated, all experiments were performed in triplicate as a minimum.

## Results

### Identification of a novel *Fgf-10* transcription start site

**Fig. 1:**
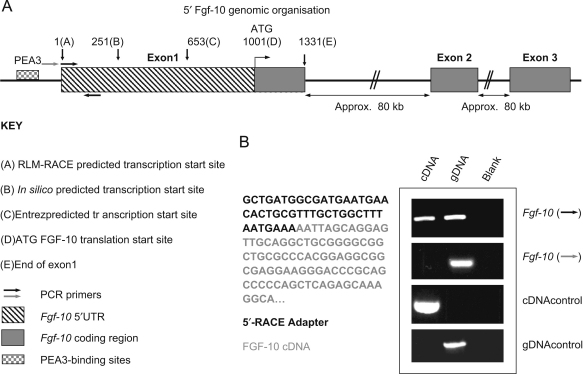
5’ Genomic organisation of *Fgf-10*. A novel *Fgf-10* transcription start site 653 nucleotides 5’ to the Entrez published site was identified by RLM-RACE using total RNA isolated from embryonic day 18.5 mouse lung (A). Analysis of the potential promoter region of *Fgf-10* (−500 to +1 bp) identified a string of 12 PEA3-binding sites approximately 360 to 240 bp upstream of the novel start site. The novel FGF-10 cDNA was confirmed by PCR analysis of cDNA and genomic (gDNA) samples (from E18.5 mouse lung) using forward primers specific to the genomic sequence immediately upstream of the predicted start site (grey) and at the predicted start of exon 1 (black), together with a common reverse primer (black) (B). PCR products of the expected size were obtained from both cDNA and gDNA samples when using the inner primer pair, confirming the predicted *Fgf-10* start site was transcribed. The gDNA primer set yielded no product with the cDNA sample, as predicted, but amplified a clear band in the gDNA sample. Primers specific for cDNA (housekeeping gene *hPRT*, with primers spanning an intron to prevent amplification of gDNA) and gDNA (known intron sequence from *Fgf-3*) confirmed the integrity of both DNA samples and the lack of gDNA contamination.

Previous studies have predicted the *Fgf-10* transcriptional start site (Katoh and Katoh, 2005), but unequivocal experimental evidence for this activity was lacking. Using total RNA from E18.5 mouse lung tissue, we performed 5’ RLM-RACE in order to identify the *Fgf-10* transcription start site. Subsequently, nested PCR was performed with specific forward primers for the 5’ RLM-RACE adaptor and reverse primers from a known sequence of the *Fgf-10* gene (data not shown). Taq-amplified 5’ RLM-RACE PCR products were cloned and four independent clones were sequenced (Fig. 1B). Here we report that we have identified a novel *Fgf-10* transcription start site, located 251 bp upstream of that predicted in silico, 653 bp upstream of the published Entrez site and 1001 bp upstream of the translation start site (Fig. 1A). Our results were confirmed by RT-PCR using a forward primer from over the newly identified transcription start which amplified a band both with cDNA generated from E18.5 mouse lung and with positive control genomic DNA (gDNA) (Fig. 1B). In contrast, RT-PCR using a forward primer recognising the 20 base pairs directly upstream of the predicted transcription start site amplified a band only with the positive control (gDNA) and not with the cDNA. Control primers, specific to an intronic sequence, confirmed the absence of genomic DNA contamination in the cDNA sample, while control primers, specific for a cDNA sequence known to be expressed (the housekeeping gene, *HPRT*), gave a strongly positive band. Thus, these data support our 5’ RLM-RACE results, suggesting a novel start site for *Fgf-10* transcription 1001 bp upstream of the translation start site.

### PEA3 binds to the *Fgf-10* promotor region

We then analysed the immediate upstream region of *Fgf-10* using an on-line Transcription Element Search System (TESS). Analysis revealed a region of approximately 120 bp in the newly defined promoter region containing 12 copies of the consensus binding sequence for PEA3 (AGGAA). This string of sites was not conserved in the human *Fgf-10* promoter, however, there were several PEA3 recognition sites within the region. Interestingly, 8 PEA3-binding sites also were present in the human (NC_000005.8) *Fgf-10* promoter region in conserved positions.

**Fig. 2:**
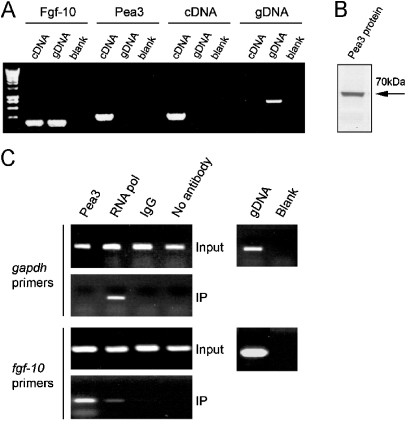
PEA3 binds to the *Fgf-10* promoter region. RT-PCR showed that immortalised murine lung endothelial cells expressed both *Fgf-10* and *Pea3* (primers as in Fig. 1 for *Fgf-10* and the cDNA and gDNA controls). *Pea3* primers were designed flanking an intron such that they amplified only cDNA (A). Western blotting of 60 μg whole-cell lysate from the same cell line showed a clear band at 70 kDa, corresponding to PEA3 (B). ChIP analysis using the same anti-PEA3 antibody showed that PEA3 binds to the *Fgf-10* promoter region (C). Immunoprecipitation with an antibody to RNA polymerase II followed by PCR using primers for *GAPDH* was used as a positive control for the ChIP assay (upper panel), with IgG and no antibody conditions used as negative controls. Presence and integrity of the ChIP input DNA was confirmed by PCR, using genomic DNA purified from ES cells as a positive control for primer efficiency. ChIP analysis using primers specific for *Fgf-10* yielded a specific PCR product both with the anti-RNA polymerase II immunoprecipitate and, more strongly, with the anti-PEA3 immunoprecipitate.

To confirm interaction of PEA3 with the *Fgf-10* promotor region, we performed ChIP experiments using immortalised mouse lung endothelial cells. RT-PCR analysis with appropriate controls confirmed that these cells expressed both FGF-10- and PEA3-encoding mRNA (Fig. 2A). Importantly, these cells expressed readily detectable PEA3 protein (Fig. 2B). An antibody to RNA polymerase II was used as a positive control for ChIP, with specific DNA binding confirmed by PCR with *GAPDH* primers. As expected, only the sample immunoprecipitated using anti-RNA polymerase II antibody yielded a PCR product (Fig. 2C, top panel). When chromatin was immunoprecipitated with antibodies to PEA3 or RNA polymerase II, *Fgf-10* sequence was detected by PCR (Fig. 2C, bottom panel). Importantly, the negative controls, where the same ChIP protocol was followed either with IgG or no antibody, gave no PCR products, suggesting that our ChIP results were a result of specific binding of RNA polymerase II to *GAPDH* and PEA3 and RNA polymerase II to *Fgf-10*, respectively. Thus, our ChIP results confirmed the binding of PEA3 to the endogenous *Fgf-10* promoter region.

### PEA3 negatively regulates *Fgf-10* expression

**Fig. 3:**
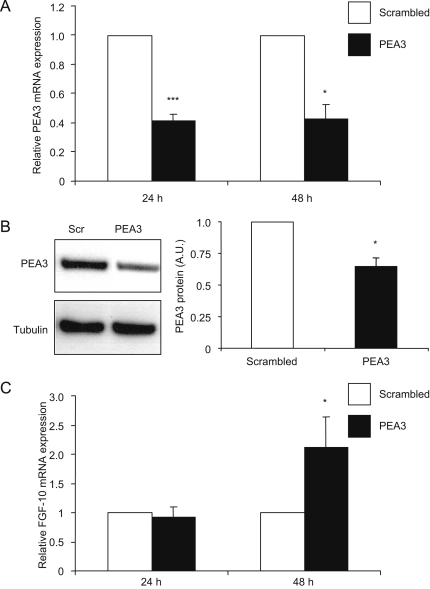
RNAi-mediated knockdown of PEA3 results in upregulation of *Fgf-10* expression. Transfection with RNAi targeted to the PEA3-coding sequence resulted in a 64% drop in *Pea3* expression levels, by 24 h and persisting at 48 h, in immortalised mouse lung endothelial cells (A). Cells transfected with a scrambled RNAi control pool showed a level of *Pea3* expression identical to that seen in mock-transfected cells (data not shown). This decrease in PEA3 mRNA level was mirrored by a decrease of 34% in the level of PEA3 protein in cell lysates prepared 48 h post-transfection (B). By 48 h post-transfection, cells treated with PEA3 RNAi showed a 2.6-fold increase in *Fgf-10* expression, as measured by real-time RT-PCR and analysed using the 2^−ΔΔCt^ method (C). All quantitation represents data from at least three independent experiments, and reflects either real-time RT-PCR data (n⩾3; each in triplicate) (A, C) or Western blot densitometry (n=3) (B). Statistical significance was determined with Student's *t*-test or Mann-Whitney Rank Sum test (PEA3 mRNA expression after 48 h). Results were considered significant at P<0.05 (*).

To determine the role for PEA3 in the regulation of *Fgf-10* expression, we used both RNAi-mediated knockdown and recombinant overexpression approaches, with subsequent real-time RT-PCR analysis. Transfection of immortalised mouse lung endothelial cells with a pool of RNAi oligos targeted specifically to PEA3 reduced the level of PEA3-encoding mRNA expression by 65% and 57%, after 24 h and 48 h, respectively, when compared to a scrambled RNAi control (Fig. 3A). By 48 h post-RNAi treatment, Western blotting of whole cell extracts showed PEA3 protein levels to be decreased by 34% (Fig. 3B). However, by 72 h post-treatment the PEA3 protein levels in RNAi-treated cells had returned to control levels (data not shown). Interestingly, although FGF-10 mRNA levels had not changed by 24 h post-RNAi treatment, after 48 h the level of FGF-10 mRNA in RNAi-treated cells was 260% higher than that of control cells (Fig. 3C). Thus, RNAi-mediated knockdown of PEA3 resulted in upregulation of FGF-10 mRNA levels, implying that PEA3 may play a negative role in the regulation of *Fgf-10* expression.

Having shown that reducing PEA3 levels led to an increase in *Fgf-10* expression, we determined whether the converse also was true. Since FGF-10 upregulation has been implicated in breast cancer (Theodorou et al., 2004), we used MDA-MB-231 and MCF-7 breast cancer cell lines as models in which to overexpress recombinant PEA3. Both cell lines expressed FGF-10 mRNA and protein as well as PEA3 mRNA, but not PEA3 protein (Supplementary Fig. 1 and data not shown). This finding was consistent with a previous study that reported, in a number of breast cancer cell lines including MDA-MB-231 and MCF-7 cells, PEA3 protein was not detectable (Baert et al., 1997).

**Fig. 4:**
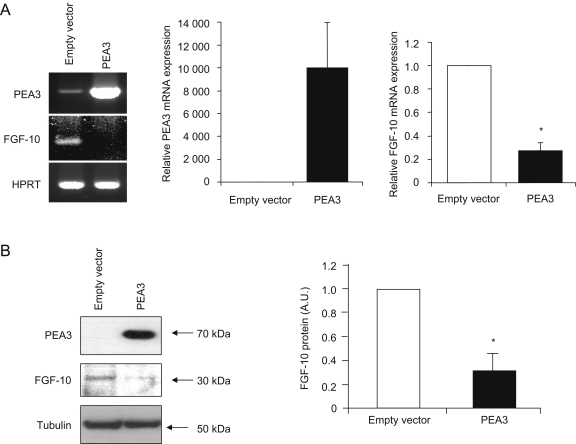
Over-expression of PEA3 in MDA-MB-231 cells decreases *Fgf-10* expression. Full-length human PEA3 cDNA (NM_001079675.1) was cloned into the pcDNA4/TO expression vector and transfected into the metastatic breast cancer cell line MDA-MB-231, with empty vector transfection serving as a control. PEA3 transfection resulted in a dramatic increase in both PEA3 mRNA (A) and protein (B) expression levels after 48 h. Concomitant with this, real-time RT-PCR showed that *Fgf-10* expression was reduced to approximately 73% of the level measured in control cells (A), and this was reflected in a similar decrease (70%) in protein levels (B). HPRT and tubulin levels were measured as loading/quality controls for mRNA and protein, respectively. Real-time RT-PCR data were analysed using the 2^−ΔΔCt^ method. All quantitation represents data from at least three independent experiments, and reflects either real-time RT-PCR data (n=5; each in triplicate) (A) or Western blot densitometry (n=3) (B). Statistical significance was determined with Student's *t*-test. Results were considered significant at P<0.05 (*).

Firstly, we cloned the full-length human PEA3-encoding cDNA sequence into a mammalian expression vector, pcDNA4/TO, and confirmed orientation and fidelity by sequencing the whole insert (data not shown). Within 48 h of transient transfection with our PEA3 expression construct, real-time RT-PCR revealed that levels of PEA3 mRNA expression in MDA-MB-231 cells increased by over 3000-fold compared to the negligible levels seen in cells transfected with empty pcDNA4/TO vector (Fig. 4A). Similarly, PEA3 protein was detected readily in PEA3-transfected MDA-MB-231 cells, but not in empty vector control cells (Fig. 4B). Crucially, at the same 48 h timepoint, PEA3-transfected cells showed a 73% reduction in FGF-10 mRNA expression relative to controls (Fig. 4A). Similar results were obtained at the protein level, with FGF-10 protein reduced by 70% (Fig. 4B). Thus, over-expression of PEA3 in MDA-MB-231 cells decreased *Fgf-10* expression. Similar results were obtained with MCF-7 cells (data not shown). These results are consistent with the RNAi-mediated knockdown of PEA3 in mouse lung endothelial cells, suggesting that PEA3 is a negative regulator of *Fgf-10* expression.

All real-time PCR results were performed using two different housekeeping genes (*HPRT* and *GAPDH*) as controls. The data presented were normalised to HPRT mRNA levels, however the results were the same when GAPDH was used for normalisation (Data not shown).

### Over-expression of PEA3 in breast cancer cells decreases cell migration

**Fig. 5:**
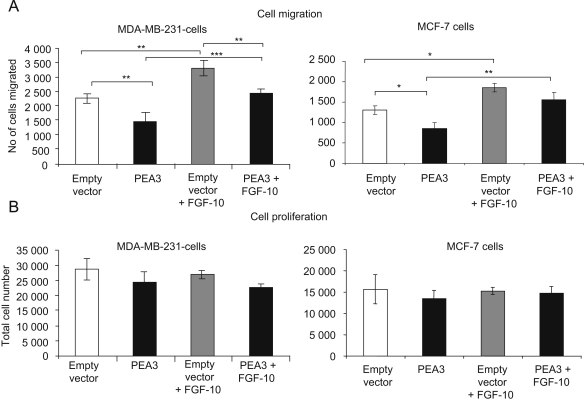
Reduction of cell migration by over-expression of PEA3 in breast cancer cells is rescued by exogenous FGF-10 treatment. MDA-MB-231 and MCF-7 cells (1×10^5^) transfected either with empty vector (white and light grey bars) or pcDNA4/TO-PEA3 (black and dark grey bars), were plated onto Transwell^®^ filters in 24-well plates 36 h post-transfection. Cells were incubated overnight in medium without FBS supplemented with 0.1% BSA and with (grey bars) or without (black and white bars) recombinant FGF-10 (100 ng/ml), where the lower surface of the membrane had been coated with fibronectin (black bars). After 12 h, for both assay conditions, significantly fewer PEA3-transfected cells had migrated to the lower surface, when compared to controls (*p<0.05; **p<0.01; ***p<0.001; ANOVA followed by Newman-Keuls test) (A). PEA3- and/or empty vector-transfected cells (5×10^4^) were plated in a 36-well plate 48 h post-transfection in serum-free medium with or without FGF-10 (100 ng/ml). Cells were trypsinised and counted 12 h after plating, revealing no significant difference between the various conditions in terms of cell proliferation (B).

In order to investigate whether PEA3 over-expression had any functional effect on breast cancer cells, we performed Transwell^®^ migration assays (Fig. 5A). Cells were allowed to migrate in the absence of FBS, since FBS contains a number of potential growth factors that might influence cell migration. The bottom of the Transwell^®^ membrane was coated with fibronectin, allowing cells migrating through the membrane to adhere to the lower surface. Interestingly, over-expression of PEA3 decreased cell migration to 62% of control levels in MDA-MB-231 cells and to 65% in MCF-7 cells (Fig. 5A). Identical results were obtained when cells were allowed to migrate in the presence of a 1–10% FBS gradient (Supplementary Fig. 2). To determine whether the inhibitory effect of PEA3 was dependent on FGF-10, we added recombinant FGF-10 (100 ng/ml) to the medium of cells transfected with a PEA3 expression construct and empty vector controls, resulting in a rescue in the migratory capacity of PEA3-expressing cells to control levels (Fig. 5A). Given that one major outcome of FGF-10 signalling is mitosis (Ornitz and Itoh, 2001), we confirmed that differences in cell proliferation did not influence our migration data. Cells in all experimental groups showed no differences in proliferation over the same timeframe that was used for our migration experiment, as expected for an assay conducted in serum-free conditions over a 12-h period (Fig. 5B). Thus, we have shown a significant inhibitory effect of PEA3 expression on cell migration and demonstrated that this reduction in migration is rescued by treatment with exogenous FGF-10.

Taken together, our results identify a novel transcription start site for *Fgf-10* and show that the transcription factor PEA3 binds upstream of this site. We go on to provide functional evidence for a role of PEA3 in negatively regulating *Fgf-10* expression, supported by RNAi knockdown, over-expression and recombinant rescue studies. From the present study, we show that PEA3 can exert an important inhibitory effect on cell migration, acting at least in part via FGF-10.

## Discussion

Despite apparently modest levels of expression, the FGF-10–FGFR2B signalling axis plays a critical role in controlling cell proliferation, apoptosis, cytoprotection and migration during embryogenesis and tissue repair (Braun et al., 2004; De Moerlooze et al., 2000; Min et al., 1998; Sekine et al., 1999). Therefore, although there are several studies reporting a tumour-suppressive effect of FGFR2 in a variety of cancer types, including bladder, prostate and skin (Feng et al., 1997; Grose and Dickson, 2005; Grose et al., 2007; Ricol et al., 1999; Zhang et al., 2001), it is easy to see how the pathway might be hijacked by cancer cells to provide them with a significant growth advantage.

Similarly to FGFR2, the literature concerning PEA3 and cancer is somewhat equivocal. Our study has shown that PEA3 overexpression in MDA-MB-231 cells decreased cell migration, and it previously has been shown that PEA3 can decrease invasion in a cervical cancer cell line (Iwasaki et al., 2004). However, the role of PEA3 in metastatic cell behaviour and cancer prognosis is contradictory. In breast cancer, PEA3 has been reported to act as a tumour suppressor gene by downregulating HER-2/neu over-expression (Xing et al., 2000). However, it also has been reported that PEA3 can play a positive role in HER2-mediated breast cancer progression (Myers et al., 2006) and that it can function as an oncogene through up-regulation of MMPs both in breast and ovarian cancer (Barrett et al., 2002; Bieche et al., 2004; Cowden Dahl et al., 2007).

Before examining regulatory elements in a gene promoter region, it clearly is essential to know the location of the true transcriptional start site. Therefore, we have identified a novel *Fgf-10* transcription start site and show that *Fgf-10* transcription is regulated by PEA3. The genomic sequence upstream of this transcription start site does not contain a canonical TATA box, however it is within a well-defined GC-rich region. The requirement for a TATA box is thought to have been overestimated since a large number of mammalian genes lack such a sequence, yet can direct accurate transcription using initiator and other downstream core promotor elements (Butler and Kadonaga, 2002; Gross and Oelgeschlager, 2006). Our study does not prove that this is the only transcriptional start site for *Fgf-10,* but it does show a novel regulatory mechanism for controlling *Fgf-10* expression. We provide clear evidence that, at least in the two breast cancer cell lines that we have used in this current study, over-expression of PEA3 has a negative influence on *Fgf-10* expression, consistent with our PEA3 knockdown data from endothelial cells, suggesting that PEA3 appears to act as a negative regulator of *Fgf-10* expression. Given the complexity of the literature regarding tumourigenic or tumour-suppressive effects of PEA3 and FGFR2B, it is possible that these effects may be cell-context dependent but the results from all the three cell lines that we have used all concur with PEA3 negatively regulating *Fgf-10* expression.

Both assay conditions that we employed to analyse cell migration, either in response to an FBS gradient or in serum-free conditions, showed that over-expression of PEA3 resulted in significant reduction in cell migration. The two assays have different merits. The FBS gradient allowed cells to migrate in a relatively undefined, but perhaps more physiologically representative, environment of various mitogenic and motogenic stimuli. Conversely, the more defined serum-free conditions were useful in determining the effect of PEA3 expression in a relatively unstimulated cell population. Regardless of their individual benefits, both assay formats gave essentially identical results. The migration assays were conducted over a relatively short timeframe of 12 h, where cell proliferation did not differ between empty vector- and PEA3-transfected cells, with or without exogenous FGF-10 treatment.

In the present study, we speculate that the inhibitory role of PEA3 on breast cancer cell migration could be due to the reduction of *Fgf-10* expression by negative regulation through PEA3. Of course, expression of genes other than *Fgf-10* will be affected by increased expression of PEA3, potentially including MMPs, uPAR and Cox-2 (Fuchs and Allgayer, 2003; Howe et al., 2001), several of which might affect cell migration. However, the rescue of the defective migration of PEA3-expressing cells by treatment with exogenous FGF-10 suggests that PEA3 acts via FGF-10. It is beyond the scope of this study to identify all the potential molecular targets that might mediate the inhibitory effect of PEA3 on the migration of MDA-MB-231 and MCF-7 cells.

**Fig. 6:**
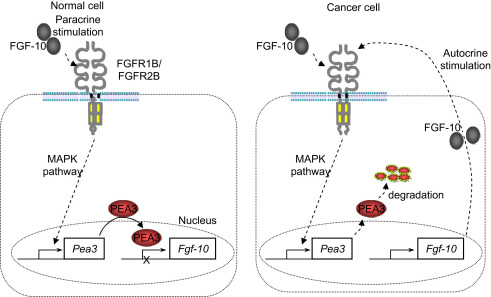
Model for PEA3-dependent regulation of FGF-10 in normal and malignant cells. In a non-malignant epithelial cell expressing FGFR1B or FGFR2B, FGF-10 secreted from a nearby cell can act in a paracrine fashion to activate its receptors and upregulate target genes, including *Pea3*. PEA3 protein can then bind to specific consensus sequences in the *Fgf-10* promoter, blocking transcription. In the cancer cells we used, PEA3 protein was unstable, meaning that it could not bind to the *Fgf-10* promoter. As a result, FGF-10 was expressed and able to stimulate its receptors in an autocrine fashion, thus providing the cancer cell with a strong growth-promoting signal.

The novel relationship between PEA3 and FGF-10 is interesting in light of previous findings that FGF-10 signalling can positively regulate *Pea3* expression during development in the mouse (Kobberup et al., 2007; Liu et al., 2003), and suggests a possible mechanism for preventing establishment of an autocrine FGF-10 positive feedback loop in cells that receive an FGF-10 signal (Fig. 6). In breast cancer cells, the lack of PEA3 could result in exactly such an autocrine situation. Thus our data suggest that the negative effect of PEA3 on cell migration in breast cancer cells warrants further investigation.

## Figures and Tables

**Table 1:** Oligonucleotides (adapters and primers) used in this study.

Method	Oligonucletotide		Sequence
5’ RLM-RACE	*Fgf-10* adapter		5’-GCUGAUGGCGAUGAAUGAACACUGCGUUUGCUGGCUUUGAUGAA
Nested PCR for 5’ RLM-RACE	Forward outer primer		5’-GCTGATGGCGATGAATGAACACTG
Forward inner primer		5’-CGCGGATCCGAACACTGCGTTTGCTGGCTTTGATG
Reverse inner primer		5’-CTGGGAACGCCAGCAGAAAAGGGA
Reverse outer primer		5’-GGTGGGACATCTGAAACCCT
Cloning of human PEA3	Forward primer^a^		5’-AT**GGATCC**AGGATGGAGCGGAGGATGAAA
Reverse primer^a^		5’-CCC**TCTAGA**CTAGTAAGAGTAGCCACCCT
RT-PCR and real-time RT-PCR	Mouse & human *HPRT*^b^	fwd	5’-CCTGCTGGATTACATTAAAGCACTG
rev	5’-GTCAAGGGCATATCCAACAACAAAC
Human *GAPDH*	fwd	5’-CCATGGAGAAGGCTGGGG
rev	5’-CAAAGTTGTCATGGATGACC
Mouse *Fgf-10*	fwd^c^	5’-CGCAATTAGCAGGAGCTGCAG
rev	5’-GGTGGGACATCTGAAACCCT
Human *Fgf-10*	fwd	5’-ATGTCCGCTGGAGAAAGCTA
rev	5’-CCTCTCCTTGGAGCTCCTTT
Human *GAPDH*^d^	fwd	5’-GACAGTCGGAAACTGGGAAG
rev	5’-GGCTGCAGGAGAAGAAAATG
Mouse *Pea3*	fwd	5’-AAACAGGAGCGCACAGACTT
rev	5’-GCCTGTCCAAGCAATGAAAT
Human *Pea3*	fwd	5’-CTGAGATCCTCTGGCACCTC
rev	5’-CTGAGTCGTAGGCGAAGTCC
Mouse *Fgf-10*^e^	fwd	5’-AATTCGGAAAGCACGCGGAC
rev	5’-GGTGGGACATCTGAAACCCT
Mouse *Fgf-3*^f^	fwd	5’-CTGTCTCACAGGATCACTAC
rev	5’-CTTGAAGCTGTAAAGGCATGC

a Engineered BamHI and XbaI recognition sites, respectively, are given in bold.

b excluding genomic DNA

c where transcription starts

d Primers in the promoter region

e directly upstream of the predicted transcription start site

f genomic DNA control primers

## Footnotes

Appendix A Supplementary data associated with this article can be found in the online version at doi:10.1016/j.ejcb.2009.01.004.
