# Supplementary figures and images for: Negative regulation of fibroblast growth factor 10 (FGF-10) by polyoma enhancer activator 3 (PEA3)
Source: Eur J Cell Biol. 2009 Jul;88(7):371–84. doi: 10.1016/j.ejcb.2009.01.004 (PMC2691923; doi:10.1016/j.ejcb.2009.01.004)

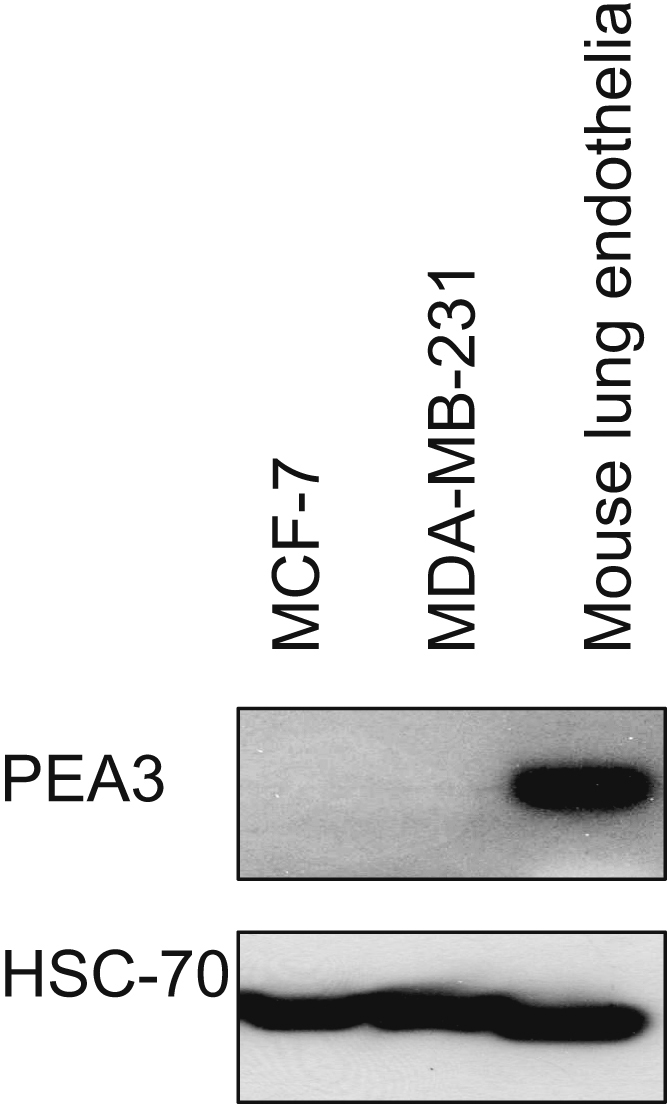

Supplement: Supplementary file 1 — Supplementary Materials [file mmc1.jpg]

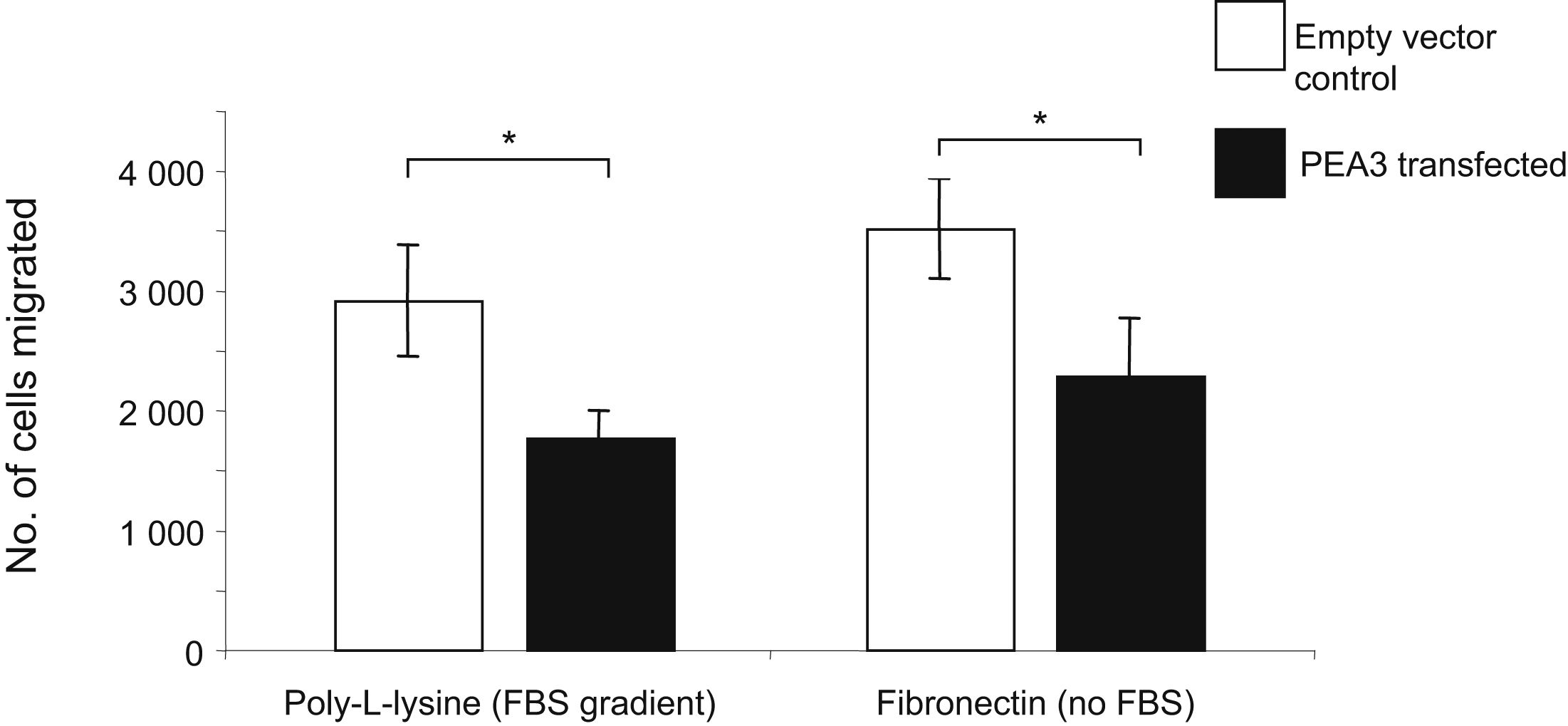

Supplement: Supplementary file 2 — Supplementary Materials [file mmc2.jpg]
